# Supplementary material for: Changes in the Incidence of Infantile Spinal Muscular Atrophy in Shikoku, Japan between 2011 and 2020
Source: Int J Neonatal Screen. 2022 Sep 26;8(4):52. doi: 10.3390/ijns8040052 (PMC9590054; doi:10.3390/ijns8040052)
Supplement: Supplementary file 1 [file IJNS-08-00052-s001.zip › File_S1.pdf]

First questionnaire

Facility number:

Does your facility currently have patients with spinal muscular atrophy who were born during 2016–2020?

(Please check one)

Yes No Unknown

In the case that the present study is published, do you agree to add your facility and the doctor's name as contributors?

( ) Yes, I agree to add the information as a contributor.

( ) No, I disagree.

( ) Other ( ).

We thank you in advance for your response.

We appreciate your continuous support.
