# Supplementary material for: Changes in the Incidence of Infantile Spinal Muscular Atrophy in Shikoku, Japan between 2011 and 2020
Source: Int J Neonatal Screen. 2022 Sep 26;8(4):52. doi: 10.3390/ijns8040052 (PMC9590054; doi:10.3390/ijns8040052)
Supplement: Supplementary file 1 [file IJNS-08-00052-s001.zip › File_S2.pdf]

Version 1.5

Second questionnaire

Re: Patients with spinal muscular atrophy who were born between January 2016 and December 2020

Please complete the information below.

Your institution anonymization number:

Patient birth month and year:

Sex: Male Female

Type of spinal muscular atrophy: type 1 type 2 other ( )

Was genetic testing used?

No Yes

(Results: copy number of *SMN1* and S*MN2*; examining institution)

Was tracheostomy with invasive positive-pressure ventilation used? No Yes

Was respiratory support needed? No Yes

Please circle ○ if the therapy was used:

Spinraza® (Starting year and month)

Zolgensma® (Starting year and month)

Evrysdi® (Starting year and month)

Present status:

Signature:

We very much appreciate your support.
