# Supplementary material for: Changes in the Incidence of Infantile Spinal Muscular Atrophy in Shikoku, Japan between 2011 and 2020
Source: Int J Neonatal Screen. 2022 Sep 26;8(4):52. doi: 10.3390/ijns8040052 (PMC9590054; doi:10.3390/ijns8040052)
Supplement: Supplementary file 1 [file IJNS-08-00052-s001.zip › Table_S1.pdf]

Table S1 Total populations of Japan and Shikoku, 2011–2020

Year	Japan			Shikoku		
	Total	Male	Female	Total	Male	Female
2011	127,799,000	62,184,000	65,615,000	3,953,000	1,874,000	2,080,000
2012	127,515,000	62,029,000	65,486,000	3,932,000	1,864,000	2,067,000
2013	127,298,000	61,909,000	65,388,000	3,905,000	1,852,000	2,053,000
2014	127,083,000	61,801,000	65,282,000	3,878,000	1,840,000	2,038,000
2015	127,095,000	61,842,000	65,253,000	3,845,000	1,829,000	2,017,000
2016	126,933,000	61,766,000	65,167,000	3,818,000	1,817,000	2,002,000
2017	126,706,000	61,655,000	65,051,000	3,788,000	1,804,000	1,984,000
2018	126,443,000	61,532,000	64,911,000	3,756,000	1,789,000	1,967,000
2019	126,167,000	61,411,000	64,756,000	3,721,000	1,774,000	1,947,000
2020	126,146,000	61,350,000	64,797,000	3,697,000	1,762,000	1,934,000

Data cited from Portal Site of Official Statistics of Japan (https://www.e-stat.go.jp/en; accessed 2022-08-11).
