# Supplementary material for: Changes in the Incidence of Infantile Spinal Muscular Atrophy in Shikoku, Japan between 2011 and 2020
Source: Int J Neonatal Screen. 2022 Sep 26;8(4):52. doi: 10.3390/ijns8040052 (PMC9590054; doi:10.3390/ijns8040052)
Supplement: Supplementary file 1 [file IJNS-08-00052-s001.zip › Table_S2.pdf]

Table S2 Live births in Japan and Shikoku, 2011–2020

Year	Japan		Shikoku	Prefecture			
	Total		Total	Ehime	Kagawa	Tokushima	Kochi
2011	1,050,807		30,798	11,329	8,311	5,914	5,244
2012	1,037,232		30,301	11,130	8,161	5,744	5,266
2013	1,029,817		29,687	10,696	8,059	5,666	5,266
2014	1,003,609		28,661	10,399	7,745	5,502	5,015
2015	1,005,721		28,503	10,146	7,719	5,586	5,052
2016	977,242		27,546	9,911	7,510	5,346	4,779
2017	946,146		26,975	9,569	7,387	5,182	4,837
2018	918,400		25,786	9,330	6,899	4,998	4,559
2019	865,239		23,901	8,446	6,631	4,554	4,270
2020	840,835		22,884	8,102	6,179	4,521	4,082

Data cited from Portal Site of Official Statistics of Japan (https://www.e-stat.go.jp/en; accessed 2022-08-11).
